# Differentiation between anatomical slenderness and acquired stenosis of the internal jugular veins

**DOI:** 10.1111/cns.13924

**Published:** 2022-08-02

**Authors:** Mengqi Wang, Xiaoqin Wu, Duo Lan, Da Zhou, Yuchuan Ding, Xunming Ji, Ran Meng

**Affiliations:** ^1^ Department of Neurology, Xuanwu Hospital Capital Medical University Beijing China; ^2^ Advanced Center of Stroke Beijing Institute for Brain Disorders Beijing China; ^3^ National Center for Neurological Disorders, Xuanwu Hospital Capital Medical University Beijing China; ^4^ Department of China‐America Institute of Neuroscience, Xuanwu Hospital Capital Medical University Beijing China; ^5^ Department of Neurosurgery Wayne State University School of Medicine Detroit Michigan USA

**Keywords:** internal jugular vein slenderness, internal jugular vein stenosis, jugular foramina, neuroimaging features

## Abstract

**Background and Purposes:**

Differentiating between acquired stenosis (pathologic) and anatomical slenderness (physiologic) of internal jugular vein (IJV) remain ambiguous. Herein, we aimed to compare the similarities and differences between the two entities.

**Methods:**

Patients who underwent head and neck computer tomography (CT) and brain magnetic resonance imaging (MRI) were enrolled in this case‐control study from January 2016 through October 2021.

**Results:**

1487 eligible patients entered final analysis totally. 803 patients had bilateral IJVs imaging without IJV stenosis‐related symptoms and presented in three ways: right IJV slenderness (10.5%, n = 85), left IJV slenderness (48.4%, *n* = 388), and symmetric IJVs (41.1%, *n* = 330). In patients with asymmetric IJVs, their bilateral jugular foramina were also asymmetric. All involved asymmetric IJVs presented as slenderness without surrounding abnormal collaterals and credible cloudy‐like white matter hyper‐intensity (WMH). Their cerebral arterial perfusion statuses on brain MR‐PWI maps were normal.

In contrast, the major patients with IJV stenosis presented with signs and symptoms such as headaches, head noise, etc. In CE‐MRV maps, local stenosis of the IJV was surrounded by abnormal venous collaterals in contrast to the lack of abnormal venous collaterals for patients with IJV slenderness. And in CTV maps, the caliber of jugular foramina was mismatched with the transverse diameter of IJV. Moreover, in MRI maps of most of these patients, a cloudy‐like WMHs were distributed symmetrically in bilateral periventricular and/or centrum semi vales. These patients also had symmetrical cerebral arterial hypo‐perfusion. Seven patients underwent stenting of the IJV stenosis correction, their WMHs attenuated or disappeared subsequently.

**Conclusions:**

Imaging features in addition to clinical symptoms can be used to differentiate between physiologic IJV slenderness and pathologic IJV stenosis. Notable imagine‐defining features for IJV stenosis include local stenosis surrounded by abnormal venous collaterals, cloudy‐like WMHs, and mismatch between the transverse diameter of IJV and the caliber of the jugular foramina.

## Graphical abstract

This study mainly illustrated the categories of anatomic modalities of internal jugular vein and the difference and similarity on neurological imaging and symptoms between slenderness and stenosis of internal jugular vein. Additionally, the effect of demographic data included gender, age and BMI and diameter of jugular foramen on slenderness of internal jugular vein were also analyzed.

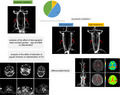

## 1 INTRODUCTION

It is well known that chronic cerebrospinal vascular insufficiency (CCSVI) is a serious disease process, often accompanied with abnormal intracranial pressure caused by cerebrovenous outflow insufficiency.^1^, ^2^, ^3^ Internal jugular vein stenosis (IJVS) has been associated with CCSVI. Recent theoretical developments have revealed that IJVS often presented with nonspecific symptoms, such as headache, head noise, tinnitus, visual impairment, neck discomfort, memory loss, and sleep disorder, etc.^4^, ^5^, ^6^, ^7^ In addition, previous observational studies have noted an association between IJVS and other neurologic diseases such as Multiple sclerosis (MS), Alzheimer's disease (AD), Leukocytosis, Migraine, Parkinson's disease (PD) and Meniere's disease.^8^, ^9^, ^10^, ^11^ Conversely, in recent decades, there has been a theory that IJVS may be a physiologic lateralization that does not need intervention. An observational study conducted by Jae Cheon Jeon et al. suggested that the diameter of the right internal jugular vein was significantly larger than that of the left in the normal population.^12^, ^13^, ^14^, ^15^, ^16^ However, many studies confirmed that IJVS had signs and symptoms, which could ameliorate or disappear after the stenosis was corrected with endovascular stenting.^5^

The variance in findings by the above authors suggested that one‐size‐fits‐all hypothesis about the internal jugular veins (IJVs) was an overgeneralization. The diametrically opposed conclusions suggests the possibility of two different processes affecting the luminal diameter of the IJV. It may be more reasonable to hypothesize that anatomical slenderness and the acquired stenosis of IJVs are two different entities that may be confused in the clinical setting.

It is necessary to provide a specific and clear answer as to whether IJV asymmetry is due to anatomical slenderness (physiologic) or acquired stenosis (pathologic). If both exist in the clinical setting, there needs to be clear delineating elements between the two modalities to facilitate a quick and accurate diagnosis of either process. These steps are necessary to improve the quality of life and long‐term outcomes of patients with IJVS. Moreover, the prevalence of anatomic asymmetric modalities of the IJV (one sided slenderness) in the Chinese population is still not clear. Herein, we analyzed the proportion of asymmetric IJVs in our single‐center Chinese patients for the first time with the help of the computer tomography venography (CTV) of head and neck and the magnetic resonance imaging (MRI) of brain and try to clarify the similarities and differences between anatomical slenderness and acquired stenosis of IJV.

## 2 METHOD AND STATISTICAL ANALYSIS

### 2.1 Patient enrollment

Eligible patients were enrolled into this case‐control study from January 1, 2016–October 1, 2021 after obtaining signed informed consents as well as an agreement from the Institutional Ethics Committee (Xuanwu Hospital, Capital Medical University). All patients underwent contrast‐enhanced neck CTV or contrast‐enhanced magnetic resonance venography (CE‐MRV) and brain MRI in our hospital. Eligible patients were divided into four groups according to the presence of IJVS‐related symptoms ‐ headache, tinnitus, visual impairment, neck discomfort, memory loss, and sleep disorders – and the appearance of the IJV on CTV maps:

1. Positive symptoms with IJV stenosis;
2. Positive symptoms without IJV stenosis;
3. Negative symptoms with IJV stenosis;
4. Negative symptoms without IJV stenosis;

Patients who underwent endovascular stenting for IJVS were followed up for 1 year.

Inclusion criteria:

1. Patients underwent head and neck CTV or CE‐MRV and brain MRI in our hospital.
2. Ages ranged from 18 to 80 years.
3. No gender preference.

Exclusion criteria:

1. Acute intracranial artery or carotid artery disease including aortic lesions, cerebral arterial disease, and congenital vascular malformation；.
2. All subtypes of cerebral venous thrombosis and non‐thrombus cerebral venous sinus stenosis；.
3. Intracranial malignant tumors；.
4. Severe hepatic or renal insufficiency and intolerance to CT or MRI by known diseases;
5. Contraindications to enhanced CT or MRI；.
6. History of head and neck cancer and previous head and neck surgery；.
7. History of previous catheter insertion in IJV；.
8. Patients who refuse to sign informed consent or did not finish CTV or CE‐MRV and MRI scan.
9. Patients with IJVS‐like symptoms could be explained by other causes.

### 2.2 Data collection

Patients without IJVS‐related symptoms and signs mentioned above were divided into four subgroups according to their age ranges: teenager (0–18 years), youth (18–45 years), middle‐aged (45‐65 years), and elderly (>65 years). These groups were subdivided into another four groups depending on BMI: underweight (<18.4 kg/m^2^), normal weight (18.5–23.9 kg/m^2^), overweight (24.0–27.9 kg/m^2^), and obesity (> = 28 kg/m^2^). Patients were also divided into two subgroups according to the gender: male and female.^16^ Clinical features related to IJVS including headache, head noise, tinnitus, visual impairment, neck discomfort, memory loss, and sleep disorder were recorded The patients relevant medical history such as hypertension, coronary heart disease, and diabetes was also recorded. All cases that meet the inclusion criteria were included in final analysis.

### 2.3 Neuroimaging assessment

Imaging studies of patients enrolled in the study were retrospectively reviewed from the inpatient medical imaging database to examine the pathological stenosis and physiological slenderness of IJVs. All patients enrolled in this study underwent MRI and CE‐MRV scanning routinely. Patients who were candidates for endovascular procedures also underwent other diagnostic modalities, such as digital subtraction angiography (DSA).

Two senior radiologists read the neck and head CTV and MR maps independently. Each radiologist calculated the cross‐sectional areas of bilateral IJVs in each patient at each segment (J1, J2, and J3). The diameters of bilateral IJVs in the same patient at the same segment were compared with each other.

In both the IJV anatomical slenderness and IJV stenosis cohorts, the clinical symptoms were compared. And the patterns of bilateral IJVs in imaging, and bilateral diameters of IJVs, collaterals and jugular foramens were also compared by means of CE‐MRV and CTV. Furthermore, some patients underwent perfusion‐weighted imaging (PWI) to evaluate the perfusion.

### 2.4 Follow‐up

All patients with IJVS obtained follow‐up imaging as well as attended outpatient clinic during the 12 ± 3 months after enrollment. Patients who underwent endovascular stenting also underwent lumber punctures at follow‐up visit. Lumbar punctures were restricted by the ICP monitoring tube, the upper limit of CSF pressure was 330 mmH2O. Lumbar puncture open pressure over 200 mmH_2_O was considered as abnormal. Clinical outcomes were graded as improvement (symptoms attenuation remarkably), no change (symptoms continue as usual), or deterioration (symptoms worsen) according the feeling of the patients themselves. Correspondingly, follow‐up neuroimaging studies of IJVS were compared with baseline studies and graded as improvement, no change, or deterioration.

### 2.5 Approvals and consents

This study was approved by the Institutional Ethics Committee of Xuanwu Hospital, Capital Medical University, Beijing, China. (Number: Clinical research 2019‐006No: 2016–006). All patients signed consent forms prior to their enrollment.

### 2.6 Statistical analysis

Descriptive statistics were calculated for all data of samples assays. All data was normally distributed, which was confirmed by the Shapiro Wilk test. All the values in the text and tables are presented as mean ‐ standard deviation (mean ± SD) and analyzed with Student t‐test. The differences of the bilateral cross‐section area of IJV were analyzed by analysis of variance. Non‐normally distributed continuous variables, as determined using the Kolmogorov–Smirnov tests were reported as medians and interquartile ranges and compared using the Mann–Whitney *U* test. Categorical variables including gender, age, body mass index (BMI) of asymptomatic cases are reported as numbers and percentages, and the effects of BMI and age as well as gender on IJVs lateralization were analyzed with chi‐square test, as appropriate. Categorical variables are analyzed with a chi‐square test. Variables were adjusted for age, sex, and BMI. The specificity and sensitivity of neuroimaging features on IJV stenosis prediction were described using Receiver‐operating characteristic curve (ROC). Two‐sided *p*‐values of <0.05 were considered statistically significant. All statistical analyses were performed using SPSS version 21.0 for Windows.

## 3 RESULTS

### 3.1 Demographics data

A total of 1640 patients were enrolled in this study. 153 patients were rejected according to exclusion criteria (41 cases had acute intracranial artery or carotid artery disease, 35 cases did not sign the informed consents, 52 cases had cerebral venous thrombosis or cerebral venous sinus stenosis, and another 25 cases had previous head or neck surgery). The 1487 eligible patients who underwent CTV were entered into final analysis according to IJVS‐related symptoms and IJV slenderness or stenosis presentation in CTV maps: 473 cases had negative symptoms but positive signs (male vs. female was 183:147), ages ranged from 14 to 85(mean 54.3 ± 14.5) years and BMI ranged from 14.7 to 35.1(mean 24.3 ± 3.6) kg/m^2^; 50 cases had positive symptoms and negative signs (male vs. female was 36:14), ages ranged from 21 to 85 (mean 57.1 ± 13.5) years and BMI ranged from 15.1 to 35.5(mean 24.7 ± 3.24) kg/m^2^; 330 cases had negative symptoms and signs (male vs. female was 305:168), ages ranged from 21 to 85(mean 55.4 ± 12.9) years and BMI ranged from 14.7 to 35.5(mean 25.3 ± 3.5) kg/m^2^; another 534 cases had positive symptoms and signs (male vs. female was 158: 376), ages ranged from 17 to 78 (mean 52.6 ± 14.57) years and BMI ranged from 17.89 to 31.23 (mean 24 ± 3.18) kg/m^2^.

### 3.2 Morphology and modalities of IJV in Chinese normal population

**FIGURE 1:**
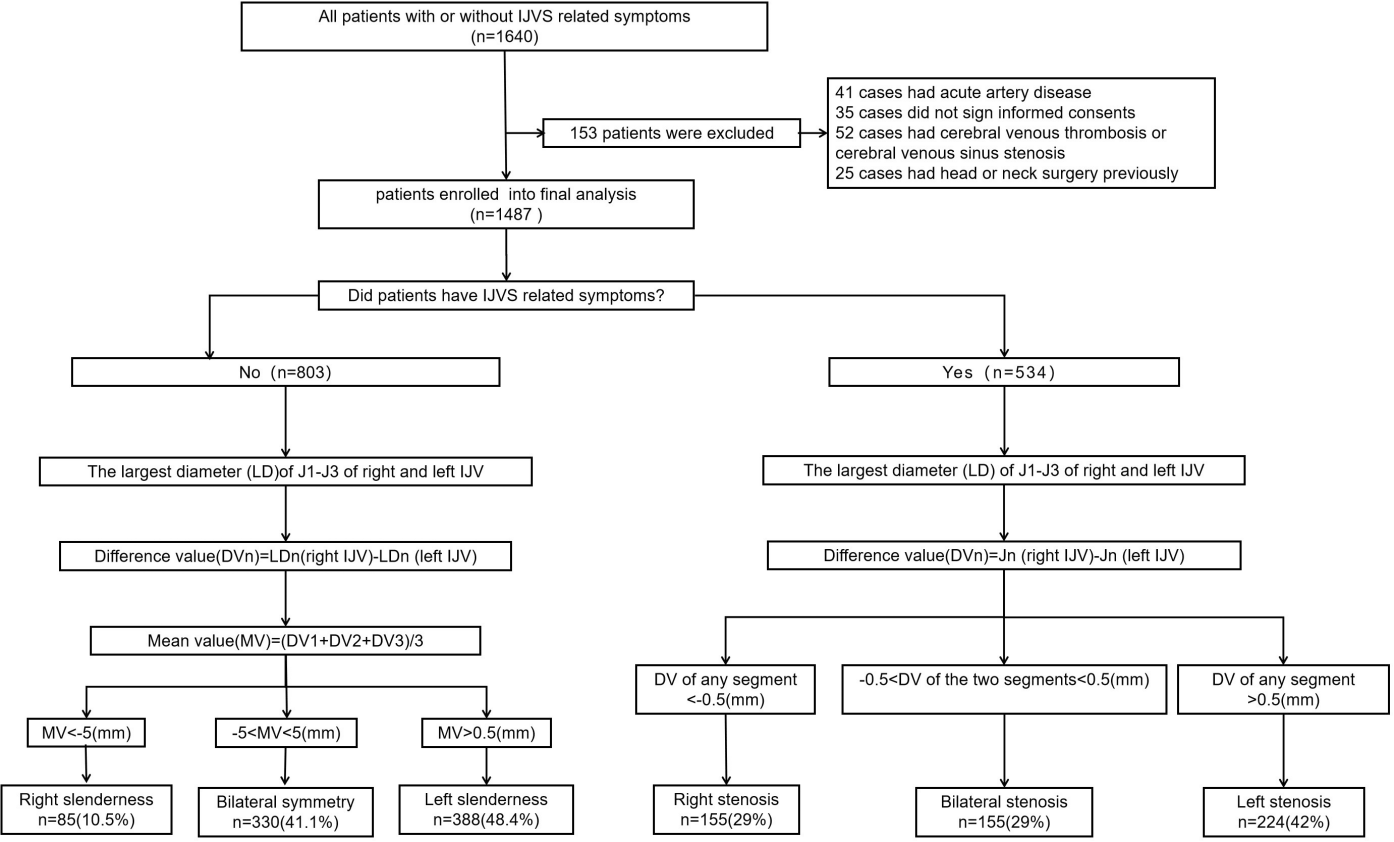
Flowchart of IJV slenderness and IJV stenosis. IJV: internal jugular vein, DV: difference value: LD: largest diameter

803 cases without IJVS‐related symptoms were analyzed. The differences of bilateral diameters of IJVs was calculated and compared at each segment of IJV (Table 1). The difference between the diameter of the two IJVs could be classified into one of three groups: right slenderness (10.5%, *n* = 85), left slenderness (48.4%, *n* = 388), and symmetry IJVs (41.1%, *n* = 330), shown in Figure 1, 2. Patient characteristics including gender, age and BMI generally did not differ among the three groups (Table 2, Figure 3).

**TABLE 1:** Comparison of the diameters of IJVs at each segment (right vs. left)

Segment	Diameter (mm)	
	Mean (SD)	Range
J1		
Right	14.55 (3.73)	7.25–28.8
Left	11.37 (2.85)	2.85–20
J2		
Right	15.08 (4.01)	4.3–24.3
Left	13.13 (3.78)	5.2–21.8
J3		
Right	10.34 (2.95)	4.8–17.9
Left	10.55 (3.58)	3.6–18.4

**FIGURE 2:**
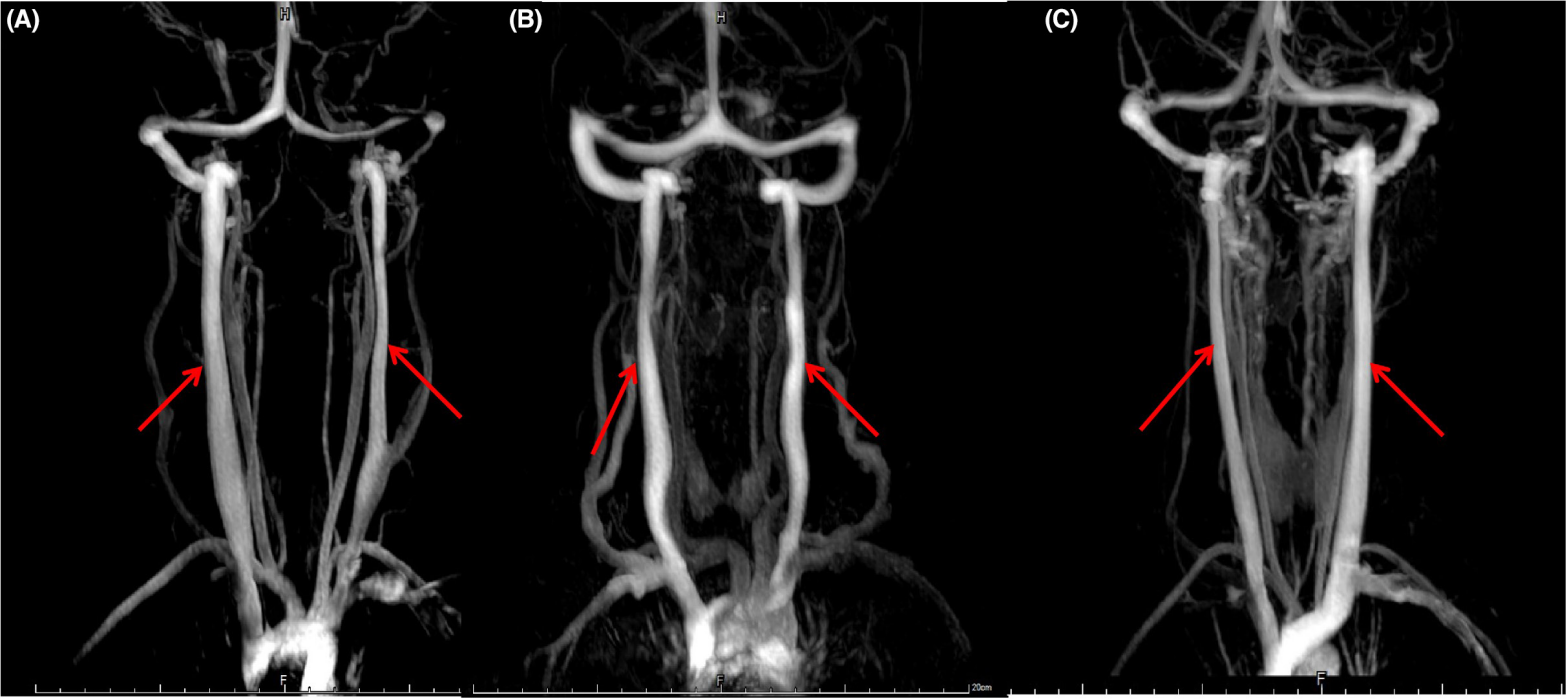
Three modalities of IJVs on CTV maps. A: right slenderness, B: bilateral symmetry, C: left slenderness

**TABLE 2:** Demographic data of patients with IJV slenderness

	Right slenderness	Bilateral symmetry	Left slenderness
Gender			
Male, *n* (%)	55(64.5%)	244(73.8%)	267(68.8%)
Female, *n* (%)	30(35.5%)	86(26.2%)	121(31.2%)
BMI			
Overweight, *n* (%)	39(45.5%)	180(54.4%)	139(35.8%)
Underweight, *n* (%)	10(11.4%)	9(2.6%)	21(5.5%)
Obesity *n* (%)	9(10.2%)	173(52.4%)	42(10.9%)
Normal weight, *n* (%)	28(33.0%)	124(37.7%)	186(47.8%)
Age			
Teenagers, *n* (%)	2(2.3%)	26(7.9%)	9(2.2%)
Youth, *n* (%)	19(22.7%)	52(15.8%)	34(8.8%)
Middle‐aged, *n* (%)	35(40.9%)	143(43.3%)	179(46.1%)
The aged, *n* (%)	29(34.1%)	110(33.3%)	166(42.9%)
Total, *n*	85	330^1^	388

**FIGURE 3:**
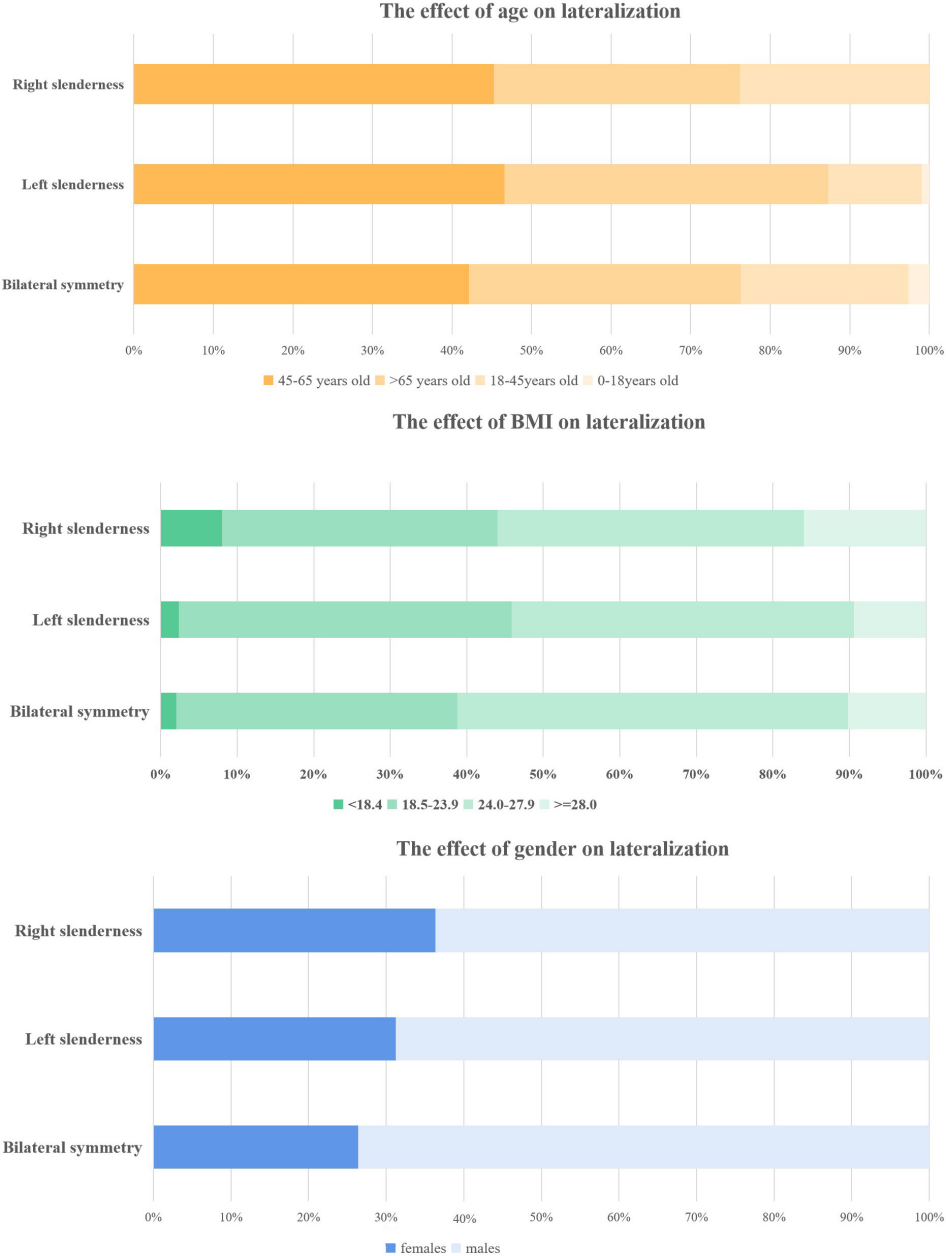
Demographic data of IJV slenderness vs. IJV stenosis

### 3.3 Neuroimaging manifestations

A total of 1006 patients were analyzed, the maps of CE‐MRV, CTV, MRI‐T2W‐FLAIR and PWI in patients with IJV slenderness (*n* = 473 without any symptoms) or IJVS (*n* = 534 with IJVS‐related symptoms) were compared as follows:

#### 3.3.1 IJV slenderness versus IJVS in CTV and/or CE‐MRV

**FIGURE 4:**
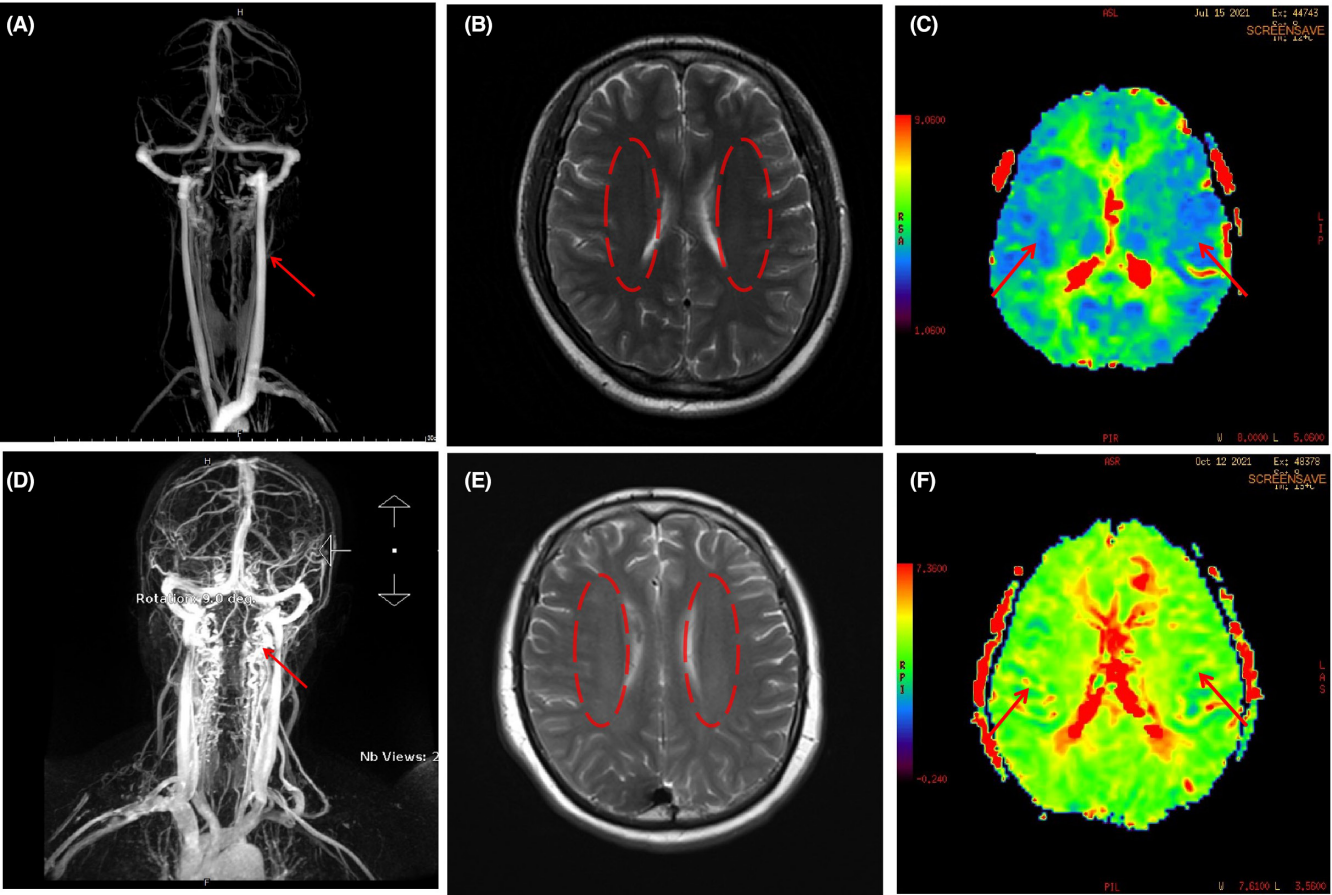
IJV slenderness and stenosis on CE‐MRV/ MRI T2W‐FLAIR /PWI. IJV slenderness (red arrow): A: CE‐MRV without surrounded collateral veins, B: MRI T2W‐FLAIR no cloudy‐like white matter hyper‐intensity (WMH), C: PWI showed normal perfusion, IJV stenosis (red arrow): D: CE‐MRV showed local stenosis surrounded by more collateral veins, E: MRI T2W‐FLAIR showed remarkable cloudy‐like WMH, F: PWI showed bilateral hypo‐perfusion

**FIGURE 5:**
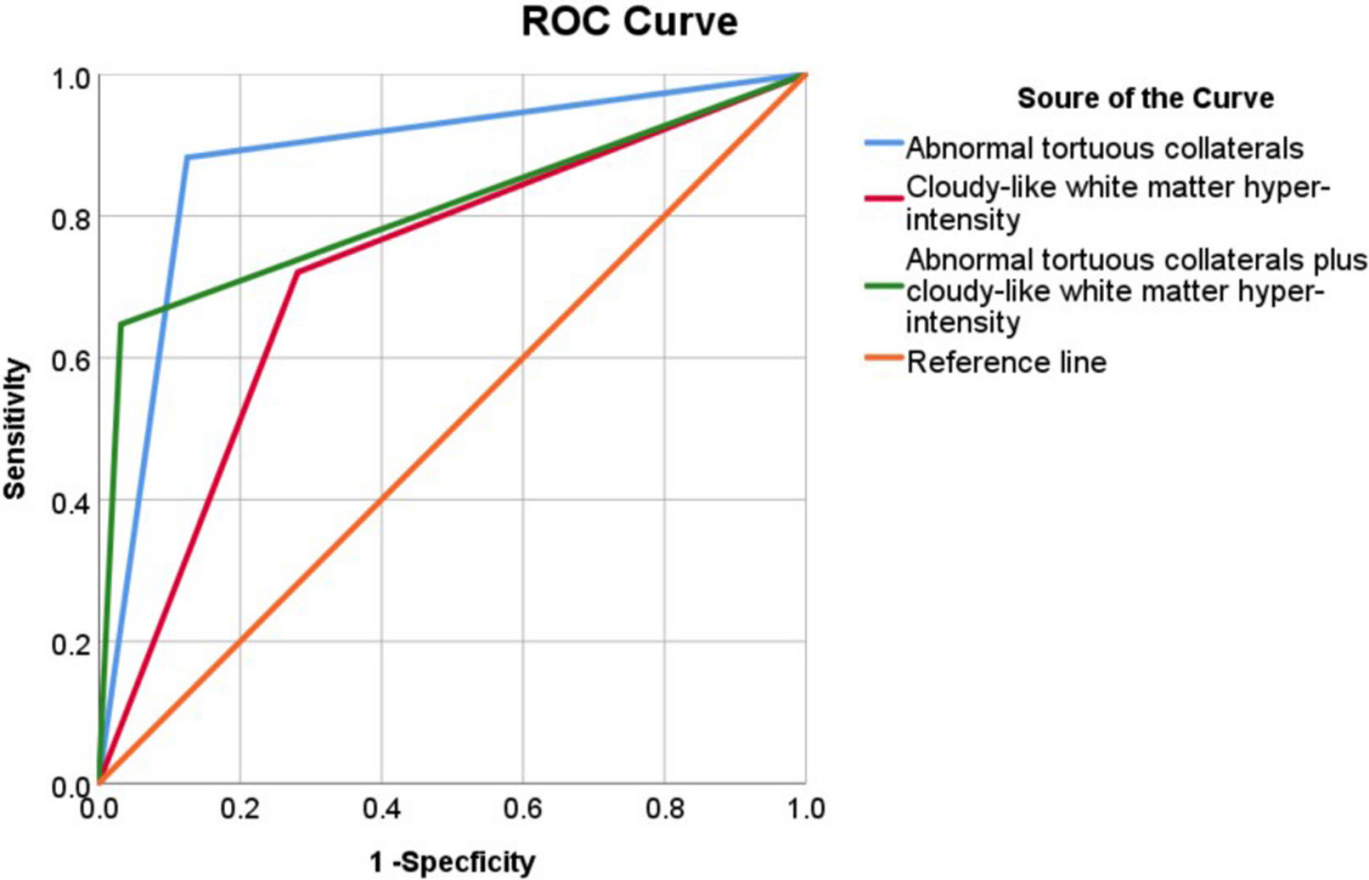
ROC curves of abnormal collaterals and cloudy‐like WMH. The blue line was ROC curve of abnormal collaterals, and the red line was the ROC curve of cloudy‐like WMH, the green line was ROC curve of abnormal collaterals and cloudy‐like WMH

From the maps of CE‐MRV and CTV, we noticed that the diameters of the involved IJV segment in both IJV slenderness and stenosis were less than that in the contralateral IJV segment. More importantly, the differences between IJV slenderness and IJVS were very remarkable. Firstly, IJV slenderness usually involved a longer segment than that of IJVS. Slenderness commonly started from the transverse sinus and sigmoid sinus and was present through the whole IJV, whereas, IJVS usually showed local stenosis of a very short segment. Secondly, patients with IJV slenderness had no or only mild collaterals (12.4%) surrounding the segment of slenderness, whereas, many patients with IJVS had numerous tortuous venous collaterals surrounding the stenotic segment (87%), *p* < 0.01, (Figure 4). The sensitivity (86.7%), specificity (87.5%), positive predicting value (87.2%), and negative predictive value (87.2%) of abnormal tortuous collaterals support its usefulness in predicting the presence of IJV acquired stenosis (Table 3). The areas under the receiver‐operating characteristic (ROC) curves of abnormal collaterals in IJV stenosis were analyzed (Figure 5).

**TABLE 3:** Sensitivity and specificity of both IJV‐surrounding tortuous collaterals and cerebral cloudy‐like white matter hyper‐intensity in the two cohorts (acquired stenosis and congenital slenderness)

Items	IJVS symptoms (*n* = 447)	None IJVS symptoms (*n* = 458)	Sensitivity (%)	Specificity (%)	Positive predictive value (%)	Negative predictive value (%)
Abnormal tortuous collaterals						
(+)	388a	57b	86.7	87.5	87.2	87.2
(−)	59c	401d				
Cloudy‐like white matter hyper‐intensity						
(+)	352a	128b	78.6	71.9	73.3	77.6
(−)	95c	330d				
Abnormal tortuous collaterals+ cloudy‐like white matter hyper‐intensity						
(+)	344a	16b	97.8	95.2	95.6	97.4
(−)	8c	298d				

*Note*: a, positive; b, false positive; c, false negative; d, true negative. IJV: internal jugular vein.

#### 3.3.2 IJV slenderness versus IJVS in MRI‐T2W‐FLAIR/PWI

A majority of patients with IJVS (78.6%) presented with demyelination changes, which frequently presented as cloudy‐like white matter hyper‐intensity(WMH)distributed at periventricular area and/or centrum semiovale on T2W‐FLAIR maps. However, in the IJV slenderness group, only a minority of patients (27.9%) showed uncertain WM，*p* < 0.01(Figure 4). The sensitivity (78.6%), specificity (71.9%), and positive predicting value (73.3%) are displayed in Table 3. Its negative predicting value was 77.6%, which can also be meaningful to predict IJVS. The areas under ROC curves were very large (Figure 5). Hemodynamic abnormality (bilateral hypo‐perfusion) could be observed clearly on PWI maps in 16 out of 20 (80%) cases with IJVS. Conversely, these appearances could not be found on PWI maps of patients with IJV slenderness. (Figure 4).

### 3.4 Symptoms and signs in patients with IJV slenderness versus IJVS

We subgrouped the patients with abnormal tortuous collaterals surrounding IJVs on CTV and/or the cloud‐like WMH on MRI. 447 patients were suspected to have IJVS, and of those 447 patients, 344 patients had both abnormal tortuous collaterals and WMH while the remaining 83 patients had neither abnormal tortuous collaterals nor WMH. 78.9% of the patients in the former subgroup had IJVS‐related symptoms, including head noise (55.7%), dizziness (42.9%), headache (42.9%), tinnitus (32.9%), visual impairment (34.3%), sleeping disorders (28.5%) and hearing loss (17.1%). While the latter group did have symptoms and signs attributable to other illnesses such as stroke, epilepsy, dementia, and arteriosclerosis, the number of patients in this group with IJVS‐related symptoms was significantly reduced. Distribution of IJVS symptoms in this group included headache (9.2%), dizziness (7.6%), and tinnitus (3.8%). Interestingly, none of them had head noise, more details were displayed in Table 4.

**TABLE 4:** Features of IJVS and IJV slenderness

	IJV stenosis (*n* = 347)	IJV slenderness (*n* = 83)	*p‐*value
Gender			
Male, *n*(%)	111(32%)	25(30%)	0.662
Female, *n*(%)	236(68%)	58(70%)	0.635
Age(years, mean ± SD), [min, max]	52.6 ± 14.57[17,78]	57.3 ± 14.6[14,85]	0.652
BMI(mean ± SD), [min, max]	24 ± 3.18 [17.89,31.23]	25.3 ± 3.5[14.7,35.5]	0.551
SYMPTOMS			
Headache, *n*(%)	149(42.9%)	8(9.2%)	<0.01*
Tinnitus, *n*(%)	114(32.9%)	4(3.8%)	<0.01*
Dizziness, *n*(%)	149(42.9%)	7(7.6%)	<0.01*
Head noise, *n*(%)	193(55.7%)	0(0)	<0.01*
Sleep disorder, *n* (%)	99(28.5%)	3(3.0%)	<0.01*
Neck discomfort, *n*(%)	45(12.9%)	9(10.0%)	0.585
Hearing loss, *n*(%)	59(17.1%)	2(1.5%)	<0.01*
Visual impairment, *n*(%)	119(34.3%)	4(4.6%)	<0.01*
Emotion abnormality, *n*(%)	59(17.1%)	3(3.1%)	<0.01*
Limb convulsion, *n*(%)	10(2.9%)	5(5.4%)	0.411
Memory loss, *n*(%)	15(4.3%)	2(2.3%)	0.434
Limb numbness, *n*(%)	15(4.3%)	2(1.5%)	0.235
Basic diseases			
Hypertension, *n*(%)	84(24.3%)	31(36.9%)	0.069
Coronary heart disease, *n*(%)	30(8.6%)	16(19.2%)	0.075
Diabetes, *n*(%)	30(8.6%)	11(13.1%)	0.341

*Note*: * indicates statistical significance as *p*‐value <0.01.

### 3.5 The size of jugular foramen in IJV slenderness versus IJVS

**FIGURE 6:**
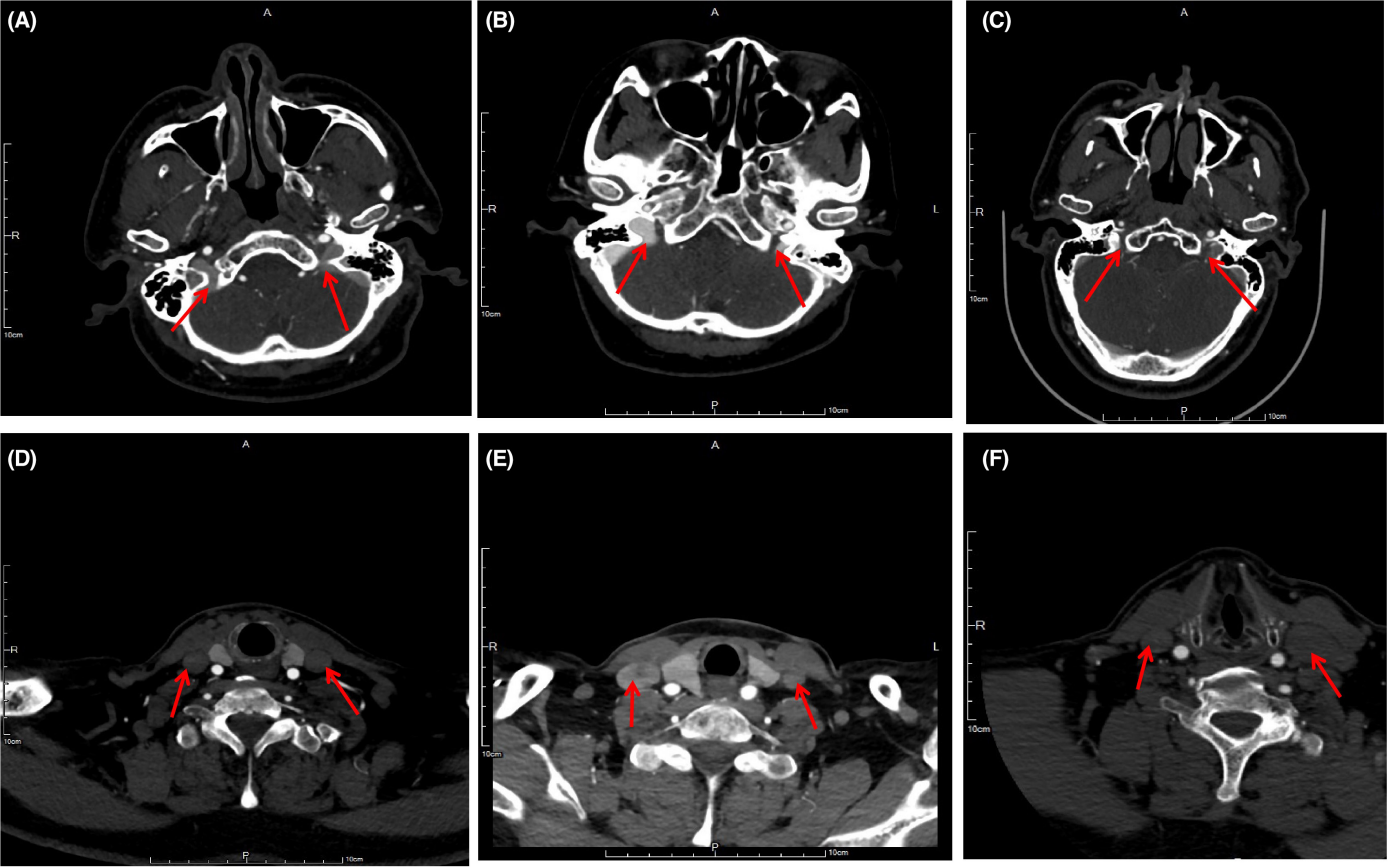
Modalities of internal jugular foramens on CT (red arrow): A: left larger‐aperture, B: right larger‐aperture, C: bilateral symmetry

The inside diameters of bilateral jugular foramens were calculated in 83 cases without IJVS and 70 cases with IJVS, the average diameter of right jugular foramens was 9.3 ± 3.46 mm, and left jugular foramens was 6.6 ± 2.85 mm. The minimum and maximum values are shown in Table 5. The differences between the mean apertures of bilateral jugular foramens can be categorized into three groups: right small, left small, and bilateral symmetry (Figure 6). Furthermore, the inside diameter group of the jugular foramens was consistent with the inside diameters group of the ipsilateral slender IJV, that is, if a patient had a slenderness of right IJV they typically also had a small right jugular foramen. However, the same pattern was not the observable in IJVS; the inside diameter of jugular foramens was not consistent with the diameters of ipsilateral stenotic IJV, that is, the inside diameters of jugular foramens were large but the ipsilateral IJV diameters were small (*p* = 0.187). Meanwhile, any routine risk factors for the difference value of aperture of bilateral jugular foramens such as gender (*p* = 0.778), BMI (*p* = 0.870), and age (*p* = 0.667) were not identified in the cases with IJVS.

**TABLE 5:** Comparison of the caliber of jugular foramen (Right vs. Left)

Side	Caliber of jugular foramen (mm)	
	Mean (SD)	Range
Right	9.3(3.46)	2.8–21.1
Left	6.6(2.85)	1.6–12.3

### 3.6 Outcomes follow‐up

Seven (4 females and 3 males) out of the 447 cases with unilateral or bilateral non‐thrombotic IJVS (right‐side in 5 cases, left‐side in 1 case and bilateral sides in 1 case) underwent stenting for the IJVS. Ages ranged from 30 to 77 (mean 50.4 ± 19.2) years, and the average follow‐up period was 12 months. All of them finished their 1‐year follow‐up.

Other intracranial pathologies such as artery anomaly, transverse sinus stenosis, and brain tumor were excluded by imaging. Among these seven patients, the most prominent IJVS‐related symptoms and signs at baseline included: headache (43.9%), head noise or tinnitus (57.1%), which was the most characteristic symptom of IJV stenosis, and visual impairment (57.1%), hearing loss (28.6%), sleep disorder (43.9%), and mildly high lumbar puncture open pressure (100%). However, all of the signs and symptoms mentioned above disappeared or significantly ameliorated post‐stenting. Four patients finished follow‐up post‐stenting imaging before discharge. Imaging showed that their blocked intravenous blood flow was completely corrected, but the WMH was still present. Four underwent post‐procedure follow‐up lumber puncture. The abnormal elevated lumbar puncture pressure in 3 cases recovered to normal post‐stenting.

In the other 440 cases of IJVS who did not undergo endovascular stenting, their symptoms did not attenuate or worsen at their 1 year follow‐up. There is still ongoing data collection about long‐term follow‐up.

## 4 DISCUSSION

Acquired stenosis (pathological) and anatomical slenderness (physiological) of the IJV are often obfuscated in clinical settings. The former needs treatment, while the later needs does not need correction. According to the published literature, this is one of the earliest studies discussing the differences between IJV congenital slenderness and acquired stenosis. The results reveal that these two entities can be distinguished by imaging and clinical symptoms. More specifically, the size of the jugular foramen and the abnormal collaterals can be used to distinguish acquired stenosis from congenital slenderness of the IJV.

This study also indicates that endovascular stenting may be a safe and efficacious treatment for correcting IJVS. In this study, seven patients underwent endovascular stenting. However, endovascular therapy is not a typical standard of treatment for IJVS. As such, it will be necessary to have larger case numbers to establish final conclusions regarding the efficacy of endovascular therapy. In addition, the larger case numbers allow for the creation of vigorous future guidelines and indications to proceed with endovascular therapy in the clinical setting.

### 4.1 Lateralization modalities and symptoms in IJV slenderness versus IJVS

This study indicated that patients with IJV anatomical slenderness seldom presented with any significant clinical symptoms. Imaging analysis of 803 patients showed that IJV slenderness could typically be grouped into one of three categories: right slenderness (10.5%), bilateral symmetry (41%), and left slenderness (48.3%). Sourava et al. also observed the lateralization of normal IJVs.^17^ Furthermore, differences in flow in the bilateral IJVs were noticed.^18^ However, due to the small sample size from the studies above, the morphological characteristics of IJVs were still not clear.

The reason for IJV slenderness formation is still unclear, but current consensus is it may be due to embryological development. Barry D. Kussman et al. found that remodeling and development during the formation of IJV during embryogenesis might be the main reason of IJV asymmetry.^19^ Contrary to the IJV slenderness, the vast majority of patients with IJV stenosis presented with long‐term symptoms of headaches, head noise, tinnitus, visual impairment, neck discomfort, memory and sleep disorders.^5^ Common causes for IJVS included local vascular wall damage, bone compression, and thrombosis^5^, ^6^.

### 4.2 IJV slenderness versus stenosis

Similarities of between slenderness and stenosis are limited; the main similarity was that the diameters of stenotic and slender IJV were significantly smaller than contralateral. Consequently, it is difficult to distinguish the imaging manifestations of these two entities on cross‐section of the CTV map. Furthermore, both IJVS and IJV slenderness could occur on any segment and any side of the IJV. These might result in misdiagnosis and incorrect treatment. As such, we suggest that a cautious yet comprehensive workup with multiple imaging perspectives and clinic features should be done before diagnosing IJV stenosis in patients. Complicating matters more is the scarcity in literature describing the difference between slenderness and stenosis of IJV. Findings in this study revealed that IJV stenosis usually involves short local segments. Whereas, IJV slenderness often involved longer segments that could extend from venous sinus till the whole IJV.^20^ Moreover, patients with IJV slenderness usually had no clinical symptom, whereas, IJVS often presented with headache, head noise, tinnitus, visual impairment, neck discomfort, memory loss, and sleep disorders.^5^, ^6^, ^21^ More importantly, abnormal venous collaterals in CE‐MRV maps and cloudy‐like WMH distributed in the periventricular area and/or centrum semiovale in MRI maps were common in IJVS rather than in IJV slenderness (Figure 4).^21^, ^22^, ^23^, ^24^ Bilateral cerebral hypo‐perfusions were also found in IJVS rather than in IJV slenderness (Figure 4).^25^

Our previous study showed that IJVS decreased the venous outflow on the stenotic side leading to compensatory collaterals formation.^5^ When the total IJV outflow (compensatory circulation and contralateral IJV) could not meet the need of the cerebral venous outflow, cerebral arterial inflow was blocked accordingly. Accordingly brain tissue ischemia happened due to stagnant deoxygenated blood in the capillaries due to venous outflow obstruction.^26^ However, the theory mentioned above may not be applicable to patients with IJV slenderness. Although slenderness resulted in cerebral venous outflow reduction in the ipsilateral IJV, the contralateral IJV could guarantee the whole cerebral outflow. Hence, the abnormal collaterals, the cloudy‐like WMH and cerebral hypo‐perfusion were not found due to intact outflow. Therefore, it stands to reason that IJVS may need intervention whereas slenderness does not.

In this study, some patients with congenital IJV slenderness still had minor abnormal collaterals surrounding the slender segment of the IJV and mild cloudy‐like WMH in the periventricular area and/or centrum semiovale. We guessed that there might be several reasons for these conditions. Firstly, most of the specific imaging changes occurred in the elderly, who had pathological arterial based changes of cerebral hypo‐perfusion or disrupted blood–brain barrier caused by arteriosclerosis, cerebral small‐vessel disease and aging‐related changes,^8^, ^9^, ^27^ Some patients had IJVS imposed on top of IJV slenderness. This appeared on imaging as venous collaterals surrounding stenotic plus slender IJV segments along with cloudy‐like WMH. Interesting enough, these patients did not have clinical symptoms. To sum up, IJV slenderness and IJVS are two different entities, one is a physiological anatomic variation and another is a pathological state, and whether IJV slenderness could cause IJVS require further researches^7^.

### 4.3 Jugular foramen caliber and IJV outer diameter

The jugular foramen is divided into pars nervosa and pars vascular and traversed by nerves and the inferior petrosal sinus, the posterior meningeal artery, and IJV.^28^, ^29^ As the bone structure is stable, the caliber of jugular foramen affects the diameter and the outflow of IJV.^30^

This study for the first time noticed that the calibers of bilateral jugular foramens were also asymmetrical in patients with unilateral IJV slenderness (*p* < 0.01), and the bilateral jugular foramens of all patients presented as three modalities in CT maps: the left larger‐caliber, the right larger‐caliber, and the bilateral symmetry. These findings were consistent with our results on the morphological characteristics of the IJV slenderness mentioned above.

Moreover, we compared the size of bilateral jugular foramens to that of bilateral IJVs in patients with either IJV slenderness or IJVS separately. We found that the sizes of the cross‐section of bilateral IJVs had a high consistency with the sizes of bilateral jugular foramens in the cohort of IJV slenderness, that is, the smaller jugular foramen was seen in the side with IJV slenderness; however, this status was different in IJVS group, the jugular foramen in the IJVS side was not smaller than that in contralateral one.

Hence, we proposed that the lateralization of jugular foramens could be as another marker to help to distinguish IJV stenosis or slenderness (Figure 6), especially in patients without significant abnormal tortuous collaterals and cloudy‐like WMH on MRI on initial stages of IJV stenosis.^5^ This study for first time illustrates internal jugular vein slenderness in detail and makes a detailed comparison between IJV stenosis and IJV slenderness, which provides a useful reference for differentiating these two processes in the clinical setting.

### 4.4 Demographics and factors affecting lateralization

In addition, we analyzed the association between demographic information (including gender, age and BMI) and IJV slenderness. The results suggested that left IJV slenderness and bilateral symmetry were more prevalent in males (69.57% and 77.27%), whereas right slenderness was more prevalent in females (80%). IJV slenderness did not appear to be related to BMI and age. Jeon, Jae Cheon et al. also mentioned the influence of gender and age on lateralization; however, Jeon, Jae Cheon et al. proposed that the outflow of the right IJV was dominant for the outflow, the consistency between our results and theirs remain to verified.^16^ The number of people less than 18 years old in our study was small, so the results may have bias in some degree.^5^

### 4.5 The efficacy and safety of endovascular stenting on correcting IJV stenosis

A previous study indicated that dehydration and medication could not be devoted to sustained improvement in the patient's symptoms from IJVS.^5^ While endovascular stenting was considered as a useful therapy on correcting abnormal elevated ICP induced by venous sinus stenosis, evidence regarding stenting to correct IJV stenosis is still lacking.^4^, ^31^, ^32^ Our study reported in 2018 confirmed that endovascular stenting could effectively correct IJV stenosis and remove IJV stenosis‐related symptoms and signs.^11^ In this study we further confirmed the effect of endovascular stenting on correcting IJV stenosis by a small self‐controlled sample in the cohort of patients with IJV stenosis. Seven cases with IJV stenosis underwent endovascular stenting and follow‐up. During the one‐year follow‐up period, all of the symptoms, signs, lumbar puncture opening pressures and venous outflow were improved significantly in almost all patients without any stenting‐related complications. However, only few of the seven patients underwent CSF test during follow‐up period. Lumbar punctures were done due to theories that CSF circulation plays an important role during the brain repair process.^33^, ^34^ However, studies with a large number of cases are still ongoing. Endovascular stenting may be a safe and effective way to improve IJV stenosis. Although CSF circulation is considered to play important role during brain repair process, studies with large number of cases are still ongoing.

## 5 LIMITATIONS

There are limitations in this study. Firstly, data of the IJVs outflow were not described besides morphology contradistinction in imaging. Secondly, less percentage of young people may cause bias to conclusion. The single‐center nature of our review as well as the homogeneity of our study population also leads to bias. It is necessary to see if the anatomic variations, imaging variations, and symptoms observed are present in people from other cities, regions, countries, and continents. Last but not least, large cases number with long‐term outcomes follow‐up are still needed along with the heterogenicity of cases as mentioned above.

## 6 CONCLUSIONS

IJV slenderness and stenosis are two different entities, which should be differentiated in clinical settings. IJVS can be differentiated from IJV slenderness by not only clinical symptoms but imaging features as well, including abnormal collaterals surrounding the IJV, symmetrically distributed cloudy‐like WMH, and the mismatch between jugular foramen aperture and IJV diameter. Some patients with IJV stenosis may get benefit from stenting. However, more studies as well as a broad selection of patients are necessary to establish guidelines and indications for placement of stents in patients with IJVS.

## AUTHOR CONTRIBUTIONS

RM involved in manuscript drafting and revision, and study concept and design. MW involved in manuscript drafting, interpretation of the data, and figure drawing. MR and MW involved in manuscript writing and final approval of the manuscript. DL, XW, DZ, and XJ involved in data collection. MR and YD involved in manuscript drafting and revision.

## FUNDING INFORMATION

This work was supported by the National Natural Science Foundation Grants (82171297); and the Beijing Natural Science Foundation (7212047).

## CONFLICT OF INTEREST

All authors reported no conflicts of interest.

## DATA AVAILABILITY STATMENT

The datasets generated during this study are available from the corresponding author upon reasonable request.

## CONSENT FOR PUBLICATION

All authors agree to publish.
